# Light-driven Proton Pumps as a Potential Regulator for Carbon Fixation in Marine Diatoms

**DOI:** 10.1264/jsme2.ME23015

**Published:** 2023-06-20

**Authors:** Susumu Yoshizawa, Tomonori Azuma, Keiichi Kojima, Keisuke Inomura, Masumi Hasegawa, Yosuke Nishimura, Masuzu Kikuchi, Gabrielle Armin, Yuya Tsukamoto, Hideaki Miyashita, Kentaro Ifuku, Takashi Yamano, Adrian Marchetti, Hideya Fukuzawa, Yuki Sudo, Ryoma Kamikawa

**Affiliations:** 1 Atmosphere and Ocean Research Institute, The University of Tokyo, Chiba 277–8564, Japan; 2 Graduate School of Frontier Sciences, The University of Tokyo, Chiba 277–8563, Japan; 3 Collaborative Research Institute for Innovative Microbiology, The University of Tokyo, Tokyo 113–8657, Japan; 4 Graduate School of Human and Environmental Studies, Kyoto University, Kyoto 606–8501, Japan; 5 School of Pharmaceutical Sciences, Okayama University, Okayama 700–8530, Japan; 6 Faculty of Medicine, Dentistry and Pharmaceutical Sciences, Okayama University, Okayama 700–8530, Japan; 7 Graduate School of Oceanography, University of Rhode Island, Narragansett, RI, USA; 8 Graduate School of Agriculture, Kyoto University, Kyoto 606–8502, Japan; 9 Graduate School of Biostudies, Kyoto University, Kyoto, 606–8502, Japan; 10 Department of Earth, Marine and Environmental Sciences, University of North Carolina at Chapel Hill, Chapel Hill, North Carolina, USA

**Keywords:** microbial rhodopsins, diatom, marine microbiology, CO_2_-concentrating mechanism

## Abstract

Diatoms are a major phytoplankton group responsible for approximately 20% of carbon fixation on Earth. They perform photosynthesis using light-harvesting chlo­rophylls located in plastids, an organelle obtained through eukaryote-eukaryote endosymbiosis. Microbial rhodopsin, a photoreceptor distinct from chlo­rophyll-based photosystems, was recently identified in some diatoms. However, the physiological function of diatom rhodopsin remains unclear. Heterologous expression techniques were herein used to investigate the protein function and subcellular localization of diatom rhodopsin. We demonstrated that diatom rhodopsin acts as a light-driven proton pump and localizes primarily to the outermost membrane of four membrane-bound complex plastids. Using model simulations, we also examined the effects of pH changes inside the plastid due to rhodopsin-mediated proton transport on photosynthesis. The results obtained suggested the involvement of rhodopsin-mediated local pH changes in a photosynthetic CO_2_-concentrating mechanism in rhodopsin-possessing diatoms.

Diatoms are unicellular, photosynthetic algae found throughout aquatic environments and are responsible for up to 20% of annual net global carbon fixation (Nelson *et al.*, 1995; Field *et al.*, 1998). Since their contribution to primary production in the ocean is significant, their light utilization mechanisms are essential to correctly understand marine ecosystems. Diatoms contain chlo­rophylls *a* and *c* and carotenoids, such as fucoxanthin, as photosynthetic pigments in the plastids acquired by eukaryote-eukaryote endosymbiosis (Keeling, 2004). Some diatoms have recently been shown to contain microbial rhodopsin (henceforth rhodopsins), a light-harvesting antenna distinct from the chlo­rophyll-containing antenna for photosynthesis (Marchetti *et al.*, 2015). Although rhodopsin-mediated light-harvesting may support the survival of these diatoms in marine environments, the physiological role of rhodopsin in diatom cells remains unclear.

Microbial rhodopsins are a large family of seven transmembrane photoreceptor proteins (Spudich *et al.*, 2000). Rhodopsin has an all-*trans* retinal as the light-absorbing chromophore, and its protein function is triggered by the light-induced isomerization of the retinal. The first microbial rhodopsin, the light-driven proton pump bacteriorhodopsin (BR), was discovered in halophilic archaea (Oesterhelt and Stoeckenius, 1971). Although rhodopsin was initially considered to only occur in halophilic archaea inhabiting hypersaline environments, subsequent studies showed that the rhodopsin gene is widely distributed in all three domains of life (Beja *et al.*, 2000; Sineshchekov *et al.*, 2002). Rhodopsins are classified based on their functions into light-driven ion pumps, light-activated signal transducers, and light-gated ion channels. The two former functional types of rhodopsin have so far been identified in prokaryotes (Ernst *et al.*, 2014). Rhodopsins in prokaryotes, regardless of function, localize to the cell membrane in which they operate. For example, proton-pumping rhodopsins export protons from the cytosol across the cell membrane to convert light energy into a proton motive force (PMF) (Yoshizawa *et al.*, 2012). The PMF induced by rhodopsin ion transport is utilized by various physiological functions, such as ATP synthesis, substrate uptake, and flagellar movement.

Rhodopsins functioning as light-driven ion pumps and light-gated ion channels have been reported in eukaryotic microorganisms (Ernst *et al.*, 2014; Kikuchi *et al.*, 2021). A light-gated ion channel called channelrhodopsin, which localizes to the plasma membrane over the eyespot within the chloroplast of green algae, has been extensively examined for its role in phototaxis (Nagel *et al.*, 2002). The other type of rhodopsin in eukaryotes, light-driven ion-pumping rhodopsins has been detected in a number of organisms belonging to both photoautotrophic and heterotrophic protists (Slamovits *et al.*, 2011; Marchetti *et al.*, 2015). Since the intracellular membrane structure of eukaryotic cells is more complex than that of prokaryotes, containing various organelles, even light-driven ion-pumping rhodopsins may have distinct physiological roles depending on their subcellular localization (Slamovits *et al.*, 2011). However, due to the difficulties associated with identifying the exact localization of rhodopsins in eukaryotic cells, their subcellular localization remains unknown.

In the present study, to clarify the physiological function of rhodopsin in a marine pennate diatom, we investigated the phylogeny, protein function, spectroscopic characteristics, and subcellular localization of rhodopsin from a member of the genus *Pseudo-nitzschia*. Heterologous expression techniques were used to analyze protein functions and spectroscopic features. The expression of rhodopsin fused with a green fluorescent protein, eGFP revealed its subcellular localization in a model diatom (*Phaeodactylum tricornutum*). Furthermore, a model-based ana­lysis was performed to evaluate the impact of the potential roles of rhodopsin in cellular biology.

## Materials and Methods

### Rhodopsin sequences and phylogenetic ana­lysis

The rhodopsin sequence of the diatom *Pseudo-nitzschia granii* was previously reported (Marchetti *et al.*, 2015). All other rhodopsin sequences used in the phylogenetic ana­lysis were collected from the National Center for Biotechnology Information. Detailed information on the strains used in this ana­lysis is given in Extended Data Fig. 1. Sequences were aligned using MAFFT version 7.453 with the options ‘—genafpair’ and ‘—maxiterate 1000’ (Katoh and Standley, 2013). The phylogenetic tree was inferred using RAxML (v.8.2.12) with the ‘PROTGAMMALGF’ model using 1,000 rapid bootstrap searches (Stamatakis, 2014). Model selection was performed with the ProteinModelSelection.pl script in the RAxML package.

The search for eukaryotic rhodopsins belonging to the Xanthorhodopsin (XR)-like rhodopsin (XLR) clade was performed among the protein sequences of the Marine Microbial Eukaryote Transcriptome Sequencing Project (MMETSP) (Keeling *et al.*, 2014). The phylogenetic placement of rhodopsin proteins from MMETSP using pplacer (v1.1.alpha19) (Matsen *et al.*, 2010) was conducted on a prebuilt large-scale phylogenetic tree of rhodopsins and extracted placements on the XLR clade using gappa (v0.6.0) (Czech *et al.*, 2020).

### Gene preparation, protein expression, and ion transport measurements of *Escherichia coli* cells

In the present study, a functional ana­lysis of the rhodopsin possessed by the diatom *P. granii* (named PngR, accession no. AJA37445.1) was performed using a heterologous expression system. The full-length cDNA for PngR, the codons of which were optimized for *E. coli*, were chemically synthesized by Eurofins Genomics and inserted into the *Nde*I-*Xho*I site of the pET21a(+) vector as previously described (Hasegawa *et al.*, 2020). A hexa-histidine-tag was fused at the C terminus of PngR, which was utilized for the purification of the expressed protein. The heterologous protein expression method is the same as that previously reported (Inoue *et al.*, 2018). *E. coli* BL21(DE3) cells harboring the cognate plasmid were grown at 37°C in LB medium supplemented with ampicillin (final concentration of 50‍ ‍μg mL^–1^). Protein expression was induced at an optical density at 600‍ ‍nm of 0.7–1.2 with 1‍ ‍mM isopropyl β-d-1-thiogalactopyranoside (IPTG) and 10‍ ‍μM all-trans retinal, after which cells were incubated at 37°C for 3 h. The proton transport activity of PngR was measured as light-induced pH changes in suspensions of *E. coli* cells as previously described (Inoue *et al.*, 2018). Briefly, cells expressing PngR were washed more than three times in 150‍ ‍mM NaCl and then resuspended in the same solution for measurements. Each cell suspension was placed in the dark for several min and then illuminated using a 300 W Xenon lamp (*ca.* 30 mW cm^–2^, MAX-303; Asahi Spectra) through a >460‍ ‍nm long-pass filter (Y48; HOYA) for 3‍ ‍min. Measurements were repeated under the same conditions after the addition of the protonophore carbonyl cyanide m-chlorophenylhydrazone (CCCP) (final concentration=10‍ ‍μM). Light-induced pH changes were monitored using a Horiba F-72 pH meter. All measurements were conducted at 25°C using a thermostat (Eyela NCB-1200; Tokyo Rikakikai).

### Purification of PngR from *E. coli* cells and spectroscopic measurements of the purified protein

*E. coli* cells expressing PngR were disrupted by sonication for 30‍ ‍min in ice-cold buffer containing 50‍ ‍mM Tris–HCl (pH 7.0) and‍ ‍300‍ ‍mM NaCl. The crude membrane fraction was collected by‍ ‍ultracentrifugation (130,000×*g* at 4°C for 60‍ ‍min) and solu­bilized with 1.0% (w/v) n-dodecyl-β-D-maltoside (DDM; DOJINDO Laboratories). The solubilized fraction was purified by Ni^2+^ affinity‍ ‍column chromatography with a linear gradient of imidazole as previously described (Kojima *et al.*, 2020b). The purified protein was concentrated by centrifugation using an Amicon Ultra filter (30,000 M_w_ cut-off; Millipore). The sample medium was then‍ ‍replaced with Buffer A (50‍ ‍mM Tris–HCl, pH 7.0, 1 M NaCl, and 0.05% [w/v] DDM) by ultrafiltration 3 times.

The absorption spectra of purified proteins were recorded using a UV-2450 spectrophotometer (Shimadzu) at room temperature in Buffer A. The retinal composition in PngR was analyzed by high-performance liquid chromatography (HPLC) as previously described (Kojima *et al.*, 2020a). Regarding dark adaptation, samples were kept under dark conditions at 4°C for more than 72 h, whereas those for light adaptation were illuminated for 3‍ ‍min at 520±10‍ ‍nm, with light power being adjusted to approximately 10‍ ‍mW‍ ‍cm^–2^. The molar compositions of the retinal isomers were calculated from the areas of the peaks in HPLC patterns monitored at 360‍ ‍nm using the extinction coefficients of retinal oxime isomers as previously described (Kojima *et al.*, 2020a). In pH titration experiments, samples were suspended in Buffer A. The pH values of the samples were adjusted to the desired acidic values by the addition of HCl, after which absorption spectra were measured at each pH value. All measurements were conducted at room temperature (approximately 25°C) under room light. After these measurements, the reversibility of spectral changes was examined to confirm that the sample was not denatured during measurements. Absorption changes at specific wavelengths were plotted against pH values and plots were fit to the Henderson–Hasselbalch equation assuming a single p*K*_a_ value as previously described (Inoue *et al.*, 2018).

The transient time-resolved absorption spectra of the purified proteins from 380 to 700‍ ‍nm at 5-nm intervals were obtained using a homemade computer-controlled flash photolysis system equipped with an Nd: YAG laser as an actinic light source. Using an optical parametric oscillator, the wavelength of the actinic pulse was tuned at 510‍ ‍nm for PngR. Pulse intensity was adjusted to 2 mJ per pulse. All data were averaged to improve the signal-to-noise ratio (*n*=30). All measurements were conducted at 25°C. In these experiments, samples were suspended in Buffer A. After measurements, the reproducibility of data was checked to confirm that the sample was not denatured during measurements. To investigate proton uptake and release during the photocycle, we used the pH indicator pyranine (final concentration=100‍ ‍μM; Tokyo Chemical Industry), which has been extensively used to monitor light-induced pH changes in various rhodopsins. pH changes in the bulk environment were measured as the absorption changes of pyranine at 450‍ ‍nm. The absorption changes of pyranine were obtained by subtracting the absorption changes of samples without pyranine from those of samples with pyranine. Experiments using pyranine were performed in an unbuffered solution containing 1 M NaCl and 0.05% (w/v) DDM (pH 7.0) to enhance signals. The results of 1,000 traces were averaged to improve the signal-to-noise ratio.

### Subcellular localization of PngR in the model diatom

The PngR:eGFP recombinant gene, coding the full length of PngR C-terminally tagged with eGFP, was cloned into the expression vector for the model diatom *P. tricornutum*, pPha-NR (Stork *et al.*, 2012), by CloneEZ (GenScript) according to the manufacturer’s instructions. The plasmid was electroporated into cells of *P. tricornutum* UTEX642 with the NEPA21 Super Electroporator (NEPAGENE), and transformed cells were selected with a Zeocin-based antibiotic treatment as previously described (Miyahara *et al.*, 2013; Dorrell *et al.*, 2019). Selected clones were observed under an Olympus BX51 fluorescent microscope (Olympus) equipped with an Olympus DP72 CCD color camera (Olympus). The nucleus stained with DAPI and chlo­rophyll autofluorescence from the plastid were observed with a 420-nm filter at 330 to 385‍ ‍nm excitation. GFP fluorescence was detected with a 510- to 550-nm filter at 470 to 495‍ ‍nm excitation.

### Quantitative model of C concentrations in a diatom: Cell Flux Model of C Concentration (CFM-CC)

*Membrane transport model.* We combined the membrane transport of CO_2_ and C fixation. Parameter definitions, units, and values are provided in Supporting Information Table S1 and S2, respectively. The key model equation is the balance of the concentration of CO_2_ in the cytosol, [*CO*_2_]*_p_*:

$$\frac{d{\left[{CO}_{2}\right]}_{p}}{dt}=D\left({\left[{CO}_{2}\right]}_{m}-{\left[{CO}_{2}\right]}_{p}\right)-V_{\text{Cfix}}\text{ [eq. 1]}$$

where *t* is time, *D* is the diffusion coefficient, and [*CO*_2_]*_m_* is the concentration of CO_2_ in the inner side of the outermost membrane of the plastid (hereafter “the middle space”). The first term represents the diffusion of CO_2_ from the middle space to the cytosol, while the second term *V_Cfix_* represents the C fixation rate following Michaelis–Menten kinetics (Berg *et al.*, 2010; Hopkinson, 2014):

$$V_{\text{Cfix}}=V_{\text{max}}\frac{{\left[{CO}_{2}\right]}_{p}}{{\left[{CO}_{2}\right]}_{p}+K}\text{ [eq. 2]}$$

where *V_max_* is the maximum CO_2_ fixation rate and *K* is the half saturation constant. [*CO*_2_]*_m_* is obtained based on the carbonate chemistry in the middle space (see below). Under the steady state, [eq. 1] with [eq. 2] becomes the following quadratic relationship for [*CO*_2_]*_p_*:

$${\left[{CO}_{2}\right]}_{p}^{2}+\left(\frac{V_{\text{max}}}{D}+K-{\left[{CO}_{2}\right]}_{m}\right){\left[{CO}_{2}\right]}_{p}-K{\left[{CO}_{2}\right]}_{m}=0\text{ [eq. 3]}$$

Solving this equation for ${\left[{CO}_{2}\right]}_{p}^{2}$ leads to:

$${\left[{CO}_{2}\right]}_{p}=\frac{-\left(\frac{V_{\text{max}}}{D}+K-{\left[{CO}_{2}\right]}_{m}\right)+\sqrt{{\left(\frac{V_{\text{max}}}{D}+K-{\left[{CO}_{2}\right]}_{m}\right)}^{2}+4K}}{2}\text{ [eq. 4]}$$

Note that the other solution for the negative route is unrealistic because it may lead to the overall negative value of [*CO*_2_]*_p_*. Once we obtain [*CO*_2_]*_p_*, we may then calculate the rate of C fixation *V_Cfix_* with [eq. 2].

Furthermore, from [eq. 4], we obtain two extreme solutions. In the case of *V_max_*≪*D* (*i.e.*, when the CO_2_ uptake capacity is small relative to the speed of CO_2_ diffusion), [eq. 4] leads to

$${\left[{CO}_{2}\right]}_{p}\;\sim \;{\left[{CO}_{2}\right]}_{m}\text{ [eq. 5]}$$

With this relationship and [eq. 2], *V_Cfix_* is computed as follows:

$$V_{\text{Cfix}}\;\sim \;V_{\text{max}}\frac{{\left[{CO}_{2}\right]}_{m}}{{\left[{CO}_{2}\right]}_{m}+K}\text{ [eq. 6]}$$

In contrast, when *V_max_*≫*D* (*i.e.*, when the CO_2_ uptake capacity is high relative to the CO_2_ diffusion across the membrane), [eq. 4] becomes

$${\left[{CO}_{2}\right]}_{p}\;\sim \;0\text{ [eq. 7]}$$

Under the steady state, [eq. 1] becomes

$$V_{\text{Cfix}}=D\left({\left[{CO}_{2}\right]}_{m}-{\left[{CO}_{2}\right]}_{p}\right)\text{ [eq. 8]}$$

and plugging [eq. 7] into [eq. 8] leads to

$$V_{\text{Cfix}}\;\sim \;D{\left[{CO}_{2}\right]}_{m}\text{ [eq. 9]}$$

and *V_Cfix_* is calculated. We note that [*CO*_2_]*_p_*>[*CO*_2_]*_m_* may occur when there are membrane-bound transporters for HCO_3_^–^ located on each membrane between the middle space and plastid (Hopkinson, 2014). However, such a set of transporters has not yet been discovered (Matsuda *et al.*, 2017). Therefore, our model conforms with the current state of knowledge. Even if [*CO*_2_]*_p_*>[*CO*_2_]*_m_*, moderately decreased pH_m_ and, thus, increased [*CO*_2_]*_m_* may be useful since they may reduce the gradient of CO_2_ across membranes (*i.e.*, [*CO*_2_]*_p_* vs [*CO*_2_]*_m_*), thereby mitigating the diffusive loss of CO_2_ from the plastid.

*Carbonate chemistry in the middle space.* The above equations may be solved once we obtain [*CO*_2_]*_m_*. The model uses a given DIC (dissolved inorganic C) concentration in the middle space [*DIC*]*_m_* to calculate [*CO*_2_]*_m_* following the established equations for carbon chemistry (Emerson and Hedges, 2008).

$${\left[{CO}_{2}\right]}_{m}=\frac{{\left[DIC\right]}_{m}}{1+\frac{K_{1}}{{\left[H^{+}\right]}_{m}}+\frac{K_{1}K_{2}}{{\left[H^{+}\right]}_{m}^{2}}}\text{ [eq. 10]}$$

where [*H*^+^]*_m_* is the concentration of H^+^ (10^–pH^ mol L^–1^) in the middle space and *K*_1_ and *K*_2_ are temperature- and salinity-dependent parameters (Lueker *et al.*, 2000; Emerson and Hedges, 2008):

$$K_{1}=10^{-pK_{1}}\text{ [eq. 11]}$$

$$K_{2}=10^{-pK_{2}}\text{ [eq. 12]}$$

where

$${pK}_{1}=\frac{3633.86}{T}-61.2172+9.6777\ln\left(T\right)-0.011555S+0.0001152S^{2}\text{ [eq. 13]}$$

$${pK}_{2}=\frac{471.78}{T}+25.9290-3.16967\ln\left(T\right)-0.01781S+0.0001122S^{2}\text{ [eq. 14]}$$

### Code availability

The code for CFM-CC is freely available from GitHub/Zenodo at https://zenodo.org/record/5182712 (DOI: 10.5281/zenodo.5182712).

## Results and Discussion

### Rhodopsin sequences and phylogenetic ana­lysis

**Fig. 1.**
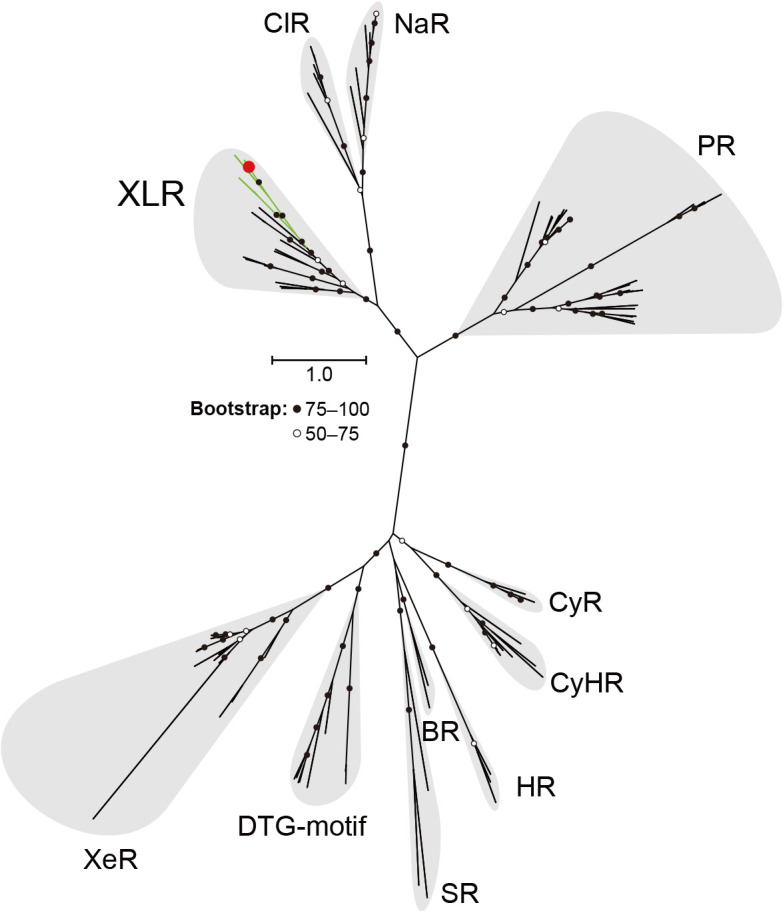
Phylogenetic position of diatom rhodopsin. A maximum likelihood tree of the amino acid sequences of microbial rhodopsins. Diatom rhodopsin (PngR) is indicated by a red circle and bootstrap probabilities (≥50%) by black and white circles. Green branches indicate eukaryotic rhodopsins used in this ana­lysis, while black branches indicate others. Rhodopsin clades are as follows: Xanthorhodopsin-like rhodopsin (XLR), Cl^–^-pumping rhodopsin (ClR), Na^+^-pumping rhodopsin (NaR), proteorhodopsin (PR), xenorhodopsin (XeR), DTG-motif rhodopsin, sensory rhodopsin-I and sensory rhodopsin-II (SR), bacteriorhodopsin (BR), halorhodopsin (HR), cyanobacterial halorhodopsin (CyHR), and cyanorhodopsin (CyR).

We performed a phylogenetic ana­lysis using the rhodopsin (named PngR, accession no. AJA37445.1) of the diatom *P. granii* and microbial rhodopsin sequences reported to date (Marchetti *et al.*, 2015). This phylogenetic tree revealed that PngR is not included in the proteorhodopsin (PR) clade commonly found in oceanic organisms, but belongs to the Xanthorhodopsin (XR)-like rhodopsin (XLR) clade, which is presumed to have an outward proton transporting function (Fig. 1 and Extended Data Fig. 1). A comparison of the motif sequences necessary for ion transport showed that the amino acids in the putative proton donor and acceptor sites of XR and PR were conserved in PngR, suggesting that PngR functions as an outward proton pump (Extended Data Fig. 2). Furthermore, the homology search for rhodopsin sequences in the XLR clade from Marine Microbial Eukaryote Transcriptome Sequencing Project (MMETSP) revealed that not only diatoms (Ochrophyta, Stramenopiles), but also dinoflagellates (Dinophyceae, Alveolata) and haptophytes have rhodopsin genes in the same XLR clade (Supporting Information Table S3). These results indicate that rhodopsins of the XLR clade are widely distributed among the major phytoplankton groups, which are important primary producers in the ocean.

### Function and spectroscopic features of diatom rhodopsin

**Fig. 2.**
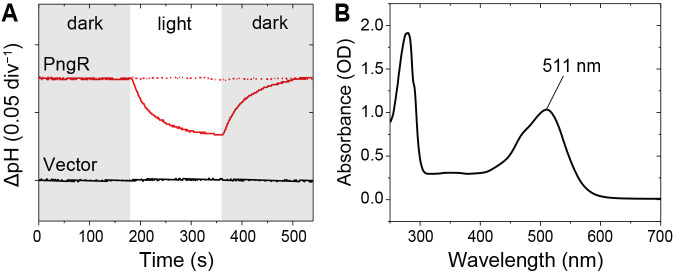
Light-induced pH changes and absorption spectrum of PngR. (A) Outward proton pump activity of PngR in *E. coli* cells. Light-induced pH changes in solutions containing *E. coli* cells with the expression plasmid for PngR (upper panel) and the empty vector pET21a (lower panel) in the presence (red dashed line) or absence (red solid line) of CCCP. The white-filled region indicates the period of illumination. (B) Absorption spectrum of purified PngR in Buffer A (50‍ ‍mM Tris–HCl, pH 7.0, 1 M NaCl, and 0.05% [w/v] DDM).

To characterize the function of PngR, we heterologously expressed the synthesized rhodopsin gene in *E. coli* cells. A light-induced decrease in pH was observed in the suspension of *E. coli* cells expressing PngR, and this reduction was almost completely abolished in the presence of the protonophore carbonyl cyanide m-chlorophenylhydrazone (CCCP) (Fig. 2A). The pH changes observed clearly showed that PngR exported protons from the cytoplasmic side across the cell membrane.

We then examined the spectroscopic characteristics of PngR using the recombinant protein purified from *E. coli*. The absorption maximum of PngR was located at 511‍ ‍nm (Fig. 2B), which was markedly shorter than those of XR (565‍ ‍nm) and GR (*Gloeobacter* rhodopsin 541‍ ‍nm) in the XLR clade (Balashov *et al.*, 2005). It is important to note that while *P. granii* is a marine species, XR and GR are both distributed in terrestrial organisms. Therefore, the present results are consistent with the shorter wavelength of the absorption maximum of rhodopsin in marine environments than in the terrestrial environment (Man *et al.*, 2003), indicating that PngR is well adapted to light conditions in the ocean, particularly the open ocean.

We then examined the retinal configuration in PngR by HPLC. In light- and dark-adapted samples, the isomeric state of retinal was predominantly all-*trans* (Extended Data Fig. 3), which was similar to the isomeric state of retinal in prokaryotic GR in the XLR clade, but different from that in BR (Miranda *et al.*, 2009). Since the p*K*_a_ value of the proton acceptor residue (Asp85 in BR) is an indicator of the efficiency of proton transport by rhodopsin, we estimated the p*K*_a_ values of the putative proton acceptor in PngR (Asp91) by a pH titration experiment (Extended Data Fig. 4). This experiment estimated that the p*K*a of this residue acceptor was approximately 5.0, indicating that the proton acceptor of PngR works well in marine and intracellular environments. Furthermore, the photochemical reactions that proceed behind the ion-transportation mechanism of PngR were examined by a flash photolysis ana­lysis (Extended Data Fig. 5). All photocycles required for ion transportation in PngR were completed in approximately 300 ms, suggesting that the cycle was sufficiently fast to pump protons in a physiologically significant time scale. The results and a discussion of the flash photolysis ana­lysis are described in the supplementary information.

### Subcellular localization of PngR in a model diatom

**Fig. 3.**
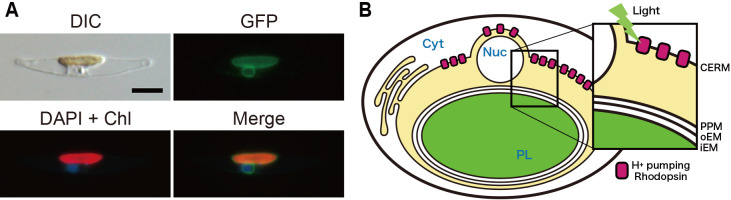
Subcellular localization of rhodopsins in diatom cells. (A) A transformed diatom cell was observed with differential interface contrast (DIC) (Upper left). Green fluorescence from recombinant PngR (GFP) (Upper right). Nuclear DNA stained with DAPI and chlo­rophyll autofluorescence (DAPI + Chl) and a merged image (Merge) are shown in the bottom left and bottom right, respectively. The scale bar indicates 5‍ ‍μm. (B) A mode for the subcellular localization of PngR. The proton transport of PngR acidifies or alkalizes the region (the middle space) surrounded by the membrane of CERM and PPM. Abbreviations are as follows: cytosol (Cyt), nucleus (Nuc), plastid (PL), chloroplast endoplasmic reticulum membrane (CERM), periplastidial membrane (PPM), outer plastid envelope membrane (oEM), and internal plastid envelope membrane (iEM).

The PngR sequence bears neither an apparent N-terminal extension nor a detectable N-terminal signal peptide, and, thus, *in silico* ana­lyses are unable to predict the subcellular localization of PngR. To identify the subcellular localization of PngR, a C-terminal eGFP-fusion PngR was expressed in the model diatom *P. tricornutum*, which may be transformed by electroporation and is often used in a heterologous expression ana­lysis (Nakajima *et al.*, 2013; Dorrell *et al.*, 2019). The transformed *P. tricornutum* cell was examined under differential interface contrast and epifluorescent microscopes (Fig. 3A). We observed the fluorescence of GFP, DAPI, and chlo­rophylls to establish the localization of recombinant PngR:eGFP, the nucleus, and chloroplast, respectively, in multiple cells (Extended Data Fig. 6 and 7). The fluorescence signal of the PngR:eGFP transformant appeared to localize at the periphery of chlo­rophyll fluorescence and DAPI signals, corresponding to the outermost plastid membrane, called the chloroplast endoplasmic reticulum membrane (CERM), which is physically connected to the nuclear membrane (Fig. 3A). A few cells also exhibited GFP signals within vacuolar membranes in addition to CERM (Extended Data Fig. 7). The insertion of the complete sequence of the PngR:eGFP gene in transformant DNA was confirmed by PCR followed by Sanger sequencing.

Based on the results of the heterologous expression experiment and microscopic observations, we concluded that PngRs primarily localized to the outermost membrane of the plastid. However, fluorescence signals were also observed to a lesser extent in the vacuolar membrane, suggesting the involvement of other factors, such as cell growth conditions, in their localization. These results imply that light-driven proton transport by PngR acidifies or alkalizes the inner region of CERM (Fig. 3B). Therefore, the physiological role of pH changes in this region in diatoms warrants further study. The electrochemical gradient formed by rhodopsin may be a driving force for various secondary transport processes. Alternatively, based on the primary purpose of plastids, local pH changes may be related to photosynthesis. The pH in this region is considered to be important for the transport of inorganic carbon (Ci) to ribulose-1,5-bisphosphate carboxylase/oxygenase (RuBisCO) (Gee and Niyogi, 2017). This is because in the carbonate system, pH affects the proportion of carbonate species (CO_2_, HCO_3_^–^, and CO_3_^2–^) in water.

Under weakly alkaline conditions in the ocean, the majority of dissolved inorganic carbon (DIC) is generally present in the form of HCO_3_^–^, with only approximately 1% being present in the form of CO_2_. However, RuBisCO localized in the stroma only reacts with Ci in the form of CO_2_, not HCO_3_^–^. The RuBisCO enzyme in diatoms exhibits low affinity even for CO_2_ (*K*m of 25~68‍ ‍μM, while CO_2_aq in the ocean is approximately 10‍ ‍μM at 25°C) and, thus, requires concentrated CO_2_ for efficient fixation at the site of RuBisCO. In other words, the ocean is always a CO_2_-limited environment for most phytoplankton (Riebesell *et al.*, 1993). Consequently, due to the membrane impermeability of HCO_3_^–^, phytoplankton have developed a number of CO_2_-concentrating mechanisms (CCM) to efficiently transport Ci to the site of RuBisCO by placing HCO_3_^–^ transporters in appropriate membranes and carbonic anhydrase (CA) in these compartments, the latter of which catalyzes the rapid interconversion between HCO_3_^–^ and CO_2_. However, since difficulties are associated with directly examining pH changes and the forms of Ci of the small compartment in eukaryotic microbial organelles, a model simulation is a powerful alternative approach (Hopkinson *et al.*, 2011). In the present study, we used a model simulation to investigate whether rhodopsin-mediated pH changes in this region were involved in CCM.

### A quantitative model of carbon concentrations in diatoms: CFM-CC

**Fig. 4.**
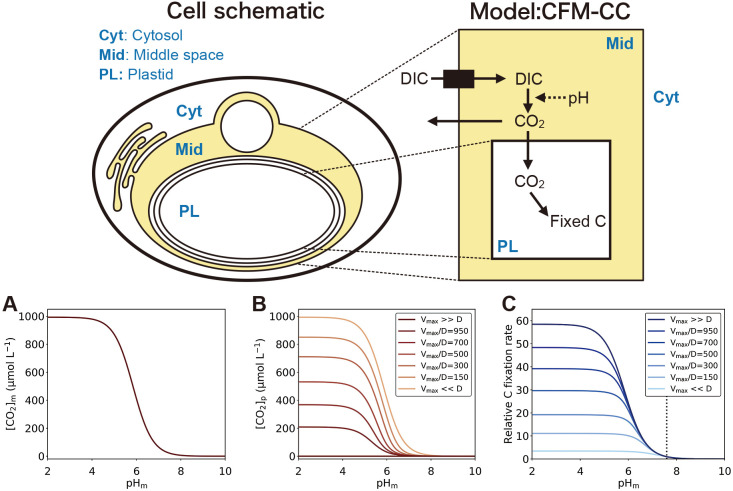
A quantitative model of the concentration of carbon in diatoms. (Upper panel) Schematic of a Cell Flux Model of C Concentration (CFM-CC). The left panel represents the actual cell, while the right panel represents the model. Solid arrows show the net flux of C and the dashed arrow indicates the effects of pH. (Bottom panel) The effects of pH in the middle space on CO_2_ concentrations and the photosynthesis rate. (A) CO_2_ concentrations in the middle space [*CO*_2_]_m_. (B) CO_2_ concentrations in the plastid [*CO*_2_]_p_. (C) The C fixation rate relative to that with pH in the middle space of 7.59, the only mean value we found for intracellular pH in a diatom (Burns and Beardall, 1987). (B) and (C) are plotted for various *V*_max_*/D*. The solution for [CO_2_]_m_ in (A) is independent of *V*_max_*/D*.

Our subcellular localization ana­lysis suggested that proton transport by rhodopsin acidified or alkalized the inner side of the outermost membrane of the plastid (the middle space). To quantitatively examine the effects of pH in the middle space on C fixation, we developed a simple quantitative model of carbonate chemistry combined with membrane transport and C fixation (CFM-CC: Cell Flux Model of C Concentration) (Fig. 4 upper panel). A comprehensive model of the concentration of CO_2_ within diatoms was developed (Hopkinson *et al.*, 2011; Hopkinson, 2014). CFM-CC uses a conceptually similar structure to this model, focusing on more specific membrane layers, designed to test the effects of pH changes in the middle space.

Our model results showed that the concentrations of CO_2_ in the middle space ([*CO*_2_]*_m_*) were strongly dependent on pH (pH_m_), suggesting that proton pumping by rhodopsin affected C fixation (Fig. 4 bottom panel). The calculation of C chemistry in the middle space revealed that a decrease in pH_m_ favored higher [*CO*_2_]*_m_* at a given DIC concentration (Fig. 4A) (we used 993‍ ‍μmol L^–1^ [Burns and Beardall, 1987]). We noted that the potential leaking of CO_2_ into the cytosol may change DIC in the middle space, but used a constant DIC value because this effect has not been experimentally demonstrated and is difficult to quantify due to unknown factors (*e.g.*, the balance of DIC uptake and CO_2_ leaking). At the reference point (we used pH_m_=7.59 [Burns and Beardall, 1987]), [*CO*_2_]*_m_* was 17‍ ‍μmol L^–1^, but increased to 64, 410, and 870‍ ‍μmol L^–1^ for pH_m_ values of 7, 6, and 5, respectively (Fig. 4A).

Due to increased [*CO*_2_]*_m_*, the concentration of CO_2_ in the plastid ([*CO*_2_]*p*) also increased with lower pH_m_; however, the level of this increase was dependent on *V*_max_*/D* (the ratio of the maximum C fixation rate to the diffusion constant) (Fig. 4B). When *V*_max_*/D* was small, the diffusion of CO_2_ from the middle space to the plastid dominated the change, resulting in [*CO*_2_]*_p_* similar to [*CO_2_*]*_m_*. In contrast, when *V*_max_*/D* was large, CO_2_ uptake dominated, and the effect of [*CO*_2_]*_m_* on [*CO*_2_]*_p_* was small.

The rate of C fixation increased with lower pH_m_ because increased [*CO*_2_]*_m_* accelerated the transport of CO_2_ into the plastid (Fig. 4C). However, the magnitude of this increase depended on *V*_max_*/D*. The model showed that the effects of pH_m_ on C fixation were greater when *V*_max_*/D* was large because the rate of C fixation was changed more by [*CO*_2_]*_m_* [eq. 6], which is directly affected by pH_m_. However, when *V*_max_*/D* was small, the C fixation rate was changed more by C uptake kinetics [eq. 4], which were saturated at a relatively low [*CO*_2_]*_p_* (*K* value of 44‍ ‍μmol L^–1^ [Jensen *et al.*, 2020]). Based on the possible range of *V_max_/D*, C fixation showed 2.1- to 3.8-, 3.2- to 24.2-, and 3.4- to 51.2-fold increases when pH_m_ decreased from 7.59 to 7, 6, 5 respectively. These results suggested that pH_m_ markedly affected C uptake at any *V*_max_*/D *as well as also the benefit for cells to have high *V*_max_ relative to the diffusivity of CO_2_ across the membrane. This was most likely the case because the *D* value was shown to be reduced when there were multiple membranes (Eichner *et al.*, 2019; Inomura *et al.*, 2019). Therefore, this simple yet elegant system with rhodopsin to manipulate pH_m_ provides a powerful mechanism in C concentrations and, thus, adjusts the C fixation rate to given physiological conditions in some rhodopsin-containing diatoms, enabling them to be more successful primary producers in the ocean.

In our model simulation, we examined the effects of rhodopsin-mediated pH changes in the middle space on CCM efficiency. The results obtained suggested that C fixation was enhanced when the pH of the middle space was acidified by a light-driven proton pump. CCM based on CO_2_ diffusion (termed the pump-leak type) has been proposed as a possible mechanism by placing CAs in appropriate locations. For example, *Nannochloropsis oceanica* (*Ochrophyta*), possessing the same four membrane-bound complex plastids as those found in diatoms, is considered to generate CO_2_ by placing CA in the middle space (Gee and Niyogi, 2017). Furthermore, the centric diatom *Chaetoceros gracilis* is considered to generate CO_2_ by placing CAs outside the cell and allowing CO_2_ to flow into the cell (Tsuji *et al.*, 2021). In contrast to the CA-based model, the acidification-based model was formerly proposed to facilitate the CO_2_ fixation of RuBisCO in the thylakoid lumen of plastids; HCO_3_^–^ is converted into CO_2_ through acidification by photosynthetic proton pumping into the thylakoid lumen (Raven, 1997). Our rhodopsin-mediated Ci transform model, CFM-CC proposes that pH changes in the middle space by proton-pumping rhodopsin also plays the role of a CO_2_ regulator. This proposed mechanism may be useful in most parts of the ocean where CO_2_ chronically limits photosynthesis, but may be even more valuable in specific environments. For example, since CA, which plays a central role in CCM, requires cobalt or zinc ions as the reaction center, and photosynthetic proton-pumping systems need iron, rhodopsin-derived acidic pools may be useful for Ci uptake in oceans where these metal ions are depleted (such as the HNLC region of the North Pacific Ocean) (Moore *et al.*, 2013). In the HNLC region of the North Pacific, where *P. granii* rhodopsin-containing cells were initially identified (Marchetti *et al.*, 2012), primary production may be limited by iron and affected by other trace metals (Saito *et al.*, 2008). In other words, our proposed mechanism appears to be particularly effective in the ocean where trace metals involved in CCM are depleted.

In the present study, we clarified the function and subcellular localization of PngR in a photosynthetic diatom. The results obtained suggest that proton transport by rhodopsin changes pH inside the outermost membrane of the plastid (CERM). A quantitative simulation indicated that the creation of an acidic pool by light provides positive feedback on C fixation efficiency, while alkalization of the middle space may restrict C fixation. If PngR acidifies the middle space, diatom rhodopsin may contribute to CCM (Extended Data Fig. 8). Future ana­lyses of cultured rhodopsin-bearing microbial eukaryotes will corroborate the present results and promote further research on the mechanisms by which rhodopsin-mediated proton transport promotes their growth in the ocean.

## Citation

Yoshizawa, S., Azuma, T., Kojima, K., Inomura, K., Hasegawa, M., Nishimura, Y., et al. (2023) Light-driven Proton Pumps as a Potential Regulator for Carbon Fixation in Marine Diatoms. *Microbes Environ ***38**: ME23015.

https://doi.org/10.1264/jsme2.ME23015

## Supplementary Material

Supplementary Material 1

Supplementary Material 2
